# Toward the unity of pathological and exertional fatigue: A predictive processing model

**DOI:** 10.3758/s13415-021-00958-x

**Published:** 2021-10-19

**Authors:** A. Greenhouse-Tucknott, J. B. Butterworth, J. G. Wrightson, N. J. Smeeton, H. D. Critchley, J. Dekerle, N. A. Harrison

**Affiliations:** 1 Fatigue and Exercise Laboratory, School of Sport and Health Sciences, University of Brighton, Brighton, East Sussex UK; 2 Department of Clinical Neurosciences, Cumming School of Medicine, University of Calgary, Calgary, AB Canada; 3 Immunopsychiatry Research Group, Cardiff University, Cardiff, UK; 4 Department of Neuroscience, Brighton and Sussex Medical School, University of Sussex, Brighton, UK; 5 Sussex Partnership NHS Foundation Trust, Brighton, UK; 6 Sackler Centre for Consciousness Science, University of Sussex, Falmer, BN1 9RR UK

**Keywords:** Fatigue, Predictive processing, Allostasis, Exercise, Interoception, Metacognition

## Abstract

Fatigue is a common experience in both health and disease. Yet, pathological (i.e., prolonged or chronic) and transient (i.e., exertional) fatigue symptoms are traditionally considered distinct, compounding a separation between interested research fields within the study of fatigue. Within the clinical neurosciences, nascent frameworks position pathological fatigue as a product of inference derived through hierarchical predictive processing. The metacognitive theory of dyshomeostasis (Stephan et al., 2016) states that pathological fatigue emerges from the metacognitive mechanism in which the detection of persistent mismatches between prior interoceptive predictions and ascending sensory evidence (i.e., prediction error) signals low evidence for internal generative models, which undermine an agent’s feeling of mastery over the body and is thus experienced phenomenologically as fatigue. Although acute, transient subjective symptoms of exertional fatigue have also been associated with increasing interoceptive prediction error, the dynamic computations that underlie its development have not been clearly defined. Here, drawing on the metacognitive theory of dyshomeostasis, we extend this account to offer an explicit description of the development of fatigue during extended periods of (physical) exertion. Accordingly, it is proposed that a loss of certainty or confidence in control predictions in response to persistent detection of prediction error features as a common foundation for the conscious experience of both pathological and nonpathological fatigue.

## Introduction

The experience of fatigue is ubiquitous in both health and disease. Although the concept of fatigue is firmly embedded within modern life, typical lay understanding of what is meant when describing oneself as fatigued can be diverse and multifaceted. Dictionary definitions of fatigue (n.) include 1) “*extreme tiredness resulting from mental or physical exertion or illness*”; 2) “*a reduction in the efficiency of a muscle or organ after prolonged activity*”; and 3) “*a lessening in one’s response to or enthusiasm for something, caused by overexposure*” (Stevenson, 2010). Thus, fatigue is often used to describe a broad combination of physical, sensory, and cognitive epiphenomena. As such, colloquial understanding of fatigue may serve to complicate its formal study, exacerbate its multidimensionality (Karshikoff et al., 2017), and emphasise the need to clearly distinguish it from other related phenomena (e.g., sleepiness) which share common descriptors (e.g., “tiredness”; Shen et al., 2006).

Currently, there is no universally accepted definition or agreed standard measure for the assessment of fatigue (Dittner et al., 2004; Kluger et al., 2013). Consequently, the study of fatigue has forged increasingly reductionistic approaches across divergent fields, fragmenting fatigue research (Pattyn et al., 2018). One common distinction is the separation of pathological fatigue from the “normal,” physiological fatigue that we all experience in everyday life. The latter is a transient, nonpathological symptom with an identifiable cause (e.g., physical or cognitive exertion), which wanes with the removal of the stressor (i.e., through rest). On the other hand, pathological fatigue is the prolonged (1-5 months) or chronic (>6 months) experience of symptoms (Jason et al., 2010), which are unalleviated by rest and may present as a primary symptom, a secondary symptom or a comorbidity within neurological diseases (Penner & Paul, 2017). Qualitative reports from patients indicate differences in both the severity and quality of fatigue experienced in disease (Flinn & Stube, 2010; Repping-Wuts et al., 2008; Scott et al., 2011) supporting this distinction. However, due to the absence of a universal and standardised measure of fatigue, quantification of these differences across health and disease may be limited by the type of fatigue assessed (e.g., trait vs. state) and the heterogeneity (e.g., uni- vs. multidimensional) of symptoms considered (Dittner et al., 2004). Indeed, the separate instruments used to assess chronic/trait versus acute symptoms of fatigue may in itself aid in perpetuating distinctions and introduce confounding affective influences (e.g., depression) on the interpretation of (trait/chronic) fatigue symptoms (c.f. Tseng et al., 2010). It remains unclear whether pathological and nonpathological fatigue can be considered fully distinct phenomena. Interestingly, a common feature of pathological fatigue is an exacerbation of symptoms following some form of acute exertion (Nijs et al., 2010). It is therefore conceivable that exertional fatigue is a fundamental component encompassed within the pathological experience of fatigue, supporting a common mechanism within both disease and health that ultimately functions to regulate energy expenditure and work output (Chaudhuri & Behan, 2004).

Within the clinical neurosciences, several theories of pathological fatigue and its underpinning neurocomputations have recently been proposed, each aligned to contemporary understanding of brain function (Kuppuswamy, 2017; Stephan et al., 2016). Building upon early propositions that perception represents unconscious, knowledge-driven inference (Helmholtz, 1860), these contemporary accounts build upon the notion that our ability to perceive, act, attend, and learn may all be accounted for by viewing the brain as a self-evidencing inference machine (Dayan et al., 1995; Friston, 2005). Predictive processing, in which explanations of sensory states are not simply extracted from sensory input streams, but instead constructed through establishing causal structures that incorporate prior expectations of sensory inputs, is posited to be a core principle of brain function (Bubic et al., 2010; Clark, 2013; Friston, 2009; Hohwy, 2014). The consideration of how precisely the brain constructs perception at the forefront of emerging accounts of pathological fatigue contrasts with many current perspectives on the transient symptoms of fatigue in response to acute, protracted exertion in health. Although previous predictive processing accounts offer a common foundation to describe and formally distinguish pathological from nonpathological, exertional fatigue (Stephan et al., 2016), an explicit account of the dynamics of the inferential processes underpinning the development of the latter is yet to be clearly described. Our goal therefore is to extend the metacognitive theory of dyshomeostasis (Stephan et al., 2016) and offer a more explicit account of the changing inferential processes that give rise to the conscious experience of fatigue during acute, transient exertion. We focus on how this framework may be applied to the study of transient fatigue symptoms arising from homeostatic perturbations incurred through sustained physical exercise.^1^

## What is Fatigue? – Fatigue vs. fatigability

Despite the absence of a standardised definition of fatigue, recent taxonomical frameworks for the study of the various causes and consequences of fatigue have been proposed (Enoka & Duchateau, 2016; Kluger et al., 2013). These taxonomies has been applied to facilitate articulation of fatigue in both neurological conditions (Kluger et al., 2013) and in response to acute, physical exertion (Enoka & Duchateau, 2016). Within it, fatigue is described as a self-reported, disabling symptom incorporating two dimensions: a subjective dimension (“perceived fatigue”), associated with broad feelings linked to weariness and exhaustion, and/or an objective dimension (“performance fatigability”), denoting a reduction in some marker of (physical and/or cognitive) performance (Kluger et al., 2013). Although these aspects of fatigue may interact, they are ultimately considered distinct and may emerge independently (Kluger et al., 2013). In the context of physical exertion, recently proposed frameworks have expanded the subjective dimension such that it includes any change in perception that aids in regulating the performer and is referred to as “perceived fatigability” (Enoka & Duchateau, 2016). Examples experienced during a task may include the perceived level of effort required (Staiano et al., 2018)and/or changes in affective valence (Hartman et al., 2019). Although combining multiple axes under one broad rubric has appeal for describing the complex and constantly changing processes involved in the control of physical performance, the concept of “perceived fatigability” may risk confusing an already complicated and multifactorial phenomenon, such as fatigue, and may detract from its functional role in the context of physical exertion. That is, the subjective component of fatigue is often conflated and used interchangeably with concepts, such as perception of effort. Although related, these represent distinct constructs (Halperin & Emanuel, 2020; Micklewright et al., 2017) with separate functional consequences (Greenhouse-Tucknott et al., 2020).

Fundamentally, fatigue represents a subjective symptom (Penner & Paul, 2017). Indeed, emphasis on changes in objective, performance-based markers of fatigue (i.e., fatigability), particularly within health, may capture only a small part of the total fatigue process since performance may be maintained through goal-orientated control processes at the expense of the perception of fatigue (Hockey, 2011; Hockey, 2013). It also is important to recognise that acute performance decrements may emerge following protracted exertion in response to other transient, affective states, such as boredom. Boredom and fatigue may be differentiated based on the level of arousal engendered by a task, corresponding to a state of under- or over-arousal, respectively (Pattyn et al., 2008). Thus, in healthy populations two conditions appear necessary for the acute, transient emergence of fatigue: 1) high task demands or arousal in which, 2) the ability to exert effective control is challenged (Hockey, 2011). In this perspective, we describe a neurocomputational account of the subjective perception of fatigue (hereafter simply referred to as fatigue) that emerges in response to changing perceptions of control efficacy during demanding physical exertion, based on a predictive processing framework.

## Hierarchical Predictive Processing – A neurobiologically plausible theory of the brain

**Fig. 1:**
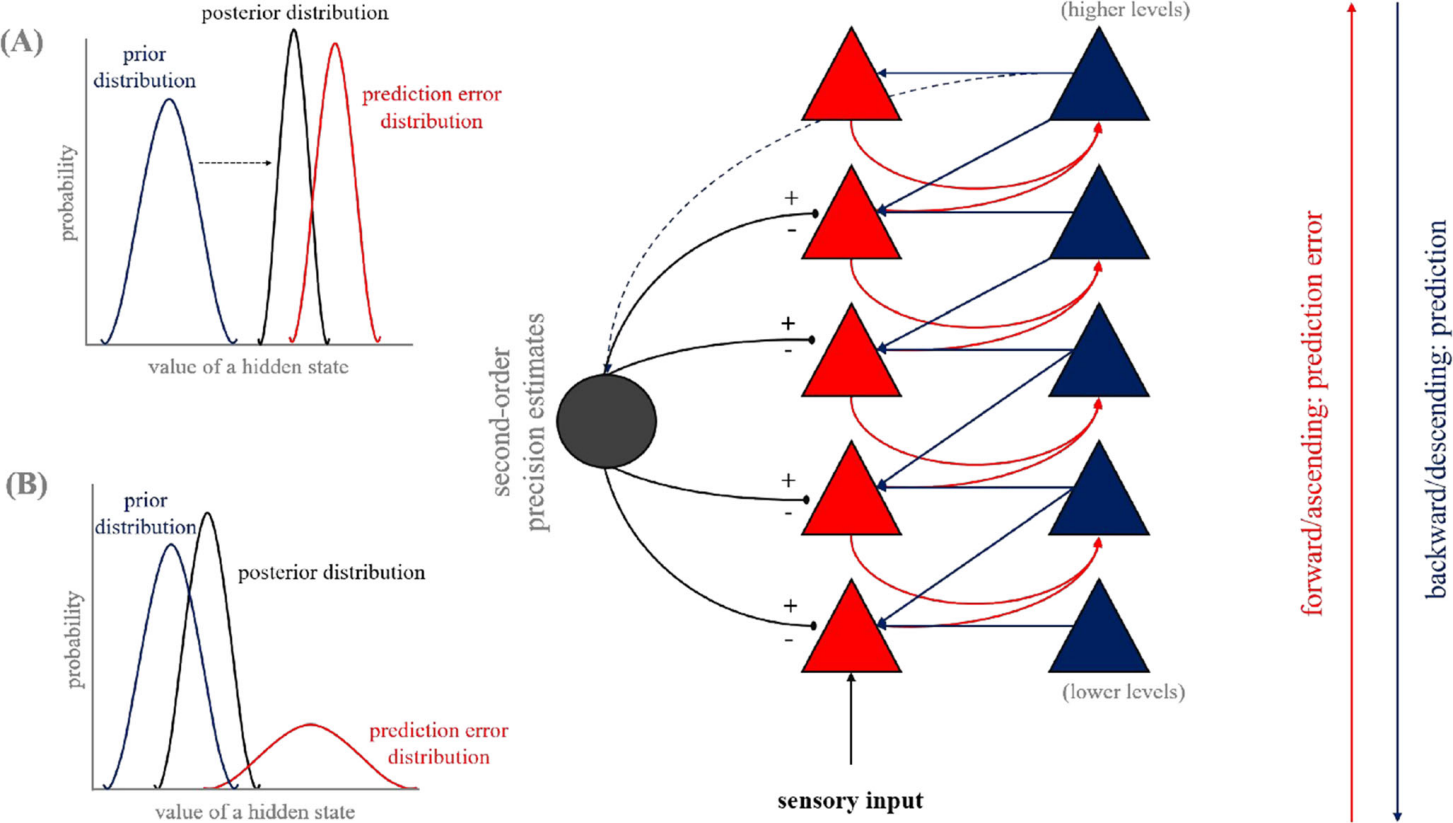
**Neuronal architecture underpinning hierarchical predictive processing.** The main figure on the right depicts a simple hierarchy that is assumed to incorporate a predictive coding encoding strategy. The system is split into five separate levels in which descending predictions (blue arrows) are transferred within the same level and to the level below. Our prior predictions are not always accurate—thus generating a prediction error. These computed prediction errors are represented by red arrows and are transferred within and between layers, ascending to the level above. The system is self-organising enabling the minimization of prediction error through updates to beliefs (i.e., posterior probability), which subsequently form new predictions passed on to the level below (i.e., empirical priors). This facilitates deep explanations of sensory inputs. Precision (i.e., inverse variance), akin to a measure of the signal-to-noise properties of an input, informs of the uncertainty or “confidence” placed in the sensory evidence. Precision determines the influence of prior beliefs relative to sensory inputs on prior updates. For example, the two depictions on the left of the figure illustrate how posterior distributions (black curves) of the value of a hidden state may be influenced by the relative precision of the prior (blue curves) and prediction error distributions (red curves). The width of the distributions indicates their variance, with precision the inverse of this variance. Precise prediction errors increase the influence of sensory evidence on updates to model predictions (i.e., posterior) (**a**). Conversely, when prediction errors are imprecise, they have little impact on the posterior belief (**b**). Precision must be estimated (second-order predictions; system not shown explicitly here) and is established by predictions descending from the highest level of the hierarchy (blue dashed line). The relative precision of prediction errors at every level of the system is believed to be controlled by neuromodulatory actions that gate or control the gain of error carrying neuronal units (grey arrows). Schematic adapted from combined works of: Ainley et al. (2016); Seth and Friston (2016)

Under conceptualisations of predictive processing, the brain is formalised as a statistical organ that continuously seeks to render the external environment predictable. This is achieved through forming and testing predictions of generative models (i.e., models that explain how sensory inputs are generated probabilistically by latent states of the world) against incoming sensory evidence (Clark, 2013). By inverting generative models (i.e., computing the probability of the states of the world, given the sensory inputs), perception is described as a form of statistical inference achieved through minimizing the discrepancy (prediction error) between predictions (or perceptual hypotheses) and current sensory evidence (Stephan et al., 2016). Unlike traditional views of perception as a stimulus-response process, perception is instead highly ingrained and contextualised by our beliefs^2^ about the statistical nature of the world (Clark, 2015). In keeping with the *Bayesian brain* hypothesis (Knill & Pouget, 2004), models are cast as following an approximate form of Bayesian inference. This inferential process is implemented through predictive coding (Friston, 2005; Rao & Ballard, 1999). Together, multilevel cortical systems are proposed to transfer prediction and prediction error across a series of recurrent ‘*loops,*’ in which probabilistic predictions (or priors/beliefs) are passed top-down from higher to lower levels, while prediction errors (i.e., the unexplained data) travel laterally (within the same level) and ascend through forward projections to the level above and serve to update predictions (Fig. 1). This is equivalent to a belief update or the transformation of prior into posterior probabilities. In these models, the goal is to update beliefs at all levels of the hierarchy such that the discrepancy between predicted states and sensory inputs is minimal. Optimization of posterior probability therefore operates through the minimization of prediction error and enables perceptual inference, which across the multiple levels of the hierarchy can provide deep explanation of sensory data (Friston, 2009; Hohwy, 2014).

Prediction error may be minimized in two ways: 1) predictions may be updated to better explain sensory inputs (i.e., perceptual inference; described above); 2) sensory evidence that conforms to prior expectations may be selectively sampled through action. For example, within the motor system, motor commands are recast as a set of proprioceptive predictions concerning the sensory consequences of action, which are enacted through classical closed-loop reflexes enacted at lower levels of the hierarchy (Adams et al., 2013). Under the framework of *active inference,* perception and action are integrated functions of a coordinated process designed to minimize the experience of prediction error (Adams et al., 2013; Friston et al., 2016). Critically, understanding how perception and action emerge within predictive frameworks also requires a measure of the expected variability in the sensory data. Therefore, inferential computations must predict the sensory inputs (first-order predictions) and the reliability of this signal (second-order predictions) to make informed judgments on which to base perception and engage action (Kanai et al., 2015). The relative precision (i.e., inverse uncertainty; reflecting signal-to-noise, or more informally, confidence in the sensory signal) of prediction and prediction error determines their respective influence on posterior beliefs (Fig. 1a and b; see Friston, 2009). Predictions of high relative precision will dominate inferential processes (e.g., action), even in the presence of conflicting sensory evidence. Conversely, predictions of low precision afford greater weighting to sensory inputs which may force us to reevaluate and update model predictions (Friston, 2009, 2010). Attention has been proposed as a process of precision inference, through which estimates of precision may be optimized (Feldman & Friston, 2010; Hohwy, 2012). Therefore attention, alongside action and perception, represents a natural component of error minimization within predictive processing. The estimation of expected precision within uncertain environments corresponds to higher-order beliefs concerning the reliability of sensory evidence (Fig. 1; Clark et al., 2018). Beliefs about beliefs (i.e., beliefs about estimated precision; hyperpriors) may provide a formalised description of metacognition (Friston et al., 2013). Within predictive processing, metacognition has been described as a high-level form of inference associated with the overall performance of prediction error minimization (Petzschner et al., 2017; Stephan et al., 2016)

## Inferred Dyshomeostasis – Allostasis, metacognition, and pathological fatigue.

As well as inferring the ambiguous states of our external environment, our brains also must infer the cause of hidden states of the body (Seth & Critchley, 2013). Several accounts have applied predictive processing within the domain of interoception to explain how dynamically changing visceral, metabolic, autonomic, immunological, and hormonal conditions are integrated (Ainley et al., 2016; Barrett, 2017; Barrett & Simmons, 2015; Gu et al., 2013; Seth et al., 2012; Seth & Critchley, 2013; Seth & Friston, 2016). All biological agents strive to maintain internal states within set bounds (homeostasis) and minimize the entropy of experienced sensory states (Friston, 2010). Interoceptive inference provides a potential explanation of how our brains respond to anticipated future needs of the body (allostasis; Sterling, 2012), and how those needs may be maintained across varying temporal scales; from (short-term) autonomic reflexes to (long-term) gross behavioural responses (Pezzulo et al., 2015). Allostasis and the representation of interoceptive consequences of action is a fundamental principle of all basic psychological functions (Kleckner et al., 2017), including our emotional experiences (Barrett, 2017; Critchley & Garfinkel, 2017; Joffily & Coricelli, 2013) and the conscious appreciation and recognition of self (Apps & Tsakiris, 2014; Seth et al., 2012; Seth & Friston, 2016). For a more in-depth discussion on the formulations of homeostatic and allostatic function within current predictive processing frameworks, see Corcoran and Hohwy (2019).

Increasingly, the (patho)aetiology of subjective feelings of fatigue have been attributed to altered computational inference, affecting the ability of hierarchical systems to reconcile sensorimotor/interoceptive predictions and sensory data (Clark et al., 2016; Edwards et al., 2012; Kuppuswamy, 2017; Manjaly et al., 2019; Stephan et al., 2016; Wrightson et al., 2019). This may occur at several loci within the hierarchy (Petzschner et al., 2017; Stephan et al., 2016). For example, dysfunctional sensory receptors (Light et al., 2009) may increase prediction error even in the absence of overt perturbations, while unduly precise prior beliefs may generate flawed perceptual inferences that are impervious to prediction error updates (van der Schaaf et al., 2018). In respect of the latter, abnormal prior beliefs endowed with high precision through the misallocation of attentional resources have been proposed as the theoretical basis of many medically unexplained or “psychogenic” symptoms (Edwards et al., 2012). In this context, pathological symptoms of fatigue may potentially emerge as an indirect consequence of abnormal attentional processes (Edwards et al., 2012). However, it remains unclear exactly how the experience of fatigue would emerge exactly. Alternative accounts have described fatigue as a direct outcome of metacognitive processing (Stephan et al., 2016). Here, continued experience of (interoceptive) prediction error, despite engaging in corrective action, is monitored within a metacognitive layer and forges the belief that allostatic control processes are incapable of effectively minimizing interceptive prediction error (that is, one experiences low “allostatic control self-efficacy”), which is experienced subjectively as fatigue (Stephan et al., 2016).

Stephan et al.'s (2016) compelling model provides a computational account of pathological fatigue, which the authors contend may be formally distinguished from the acute, transient exertional fatigue experienced in health (referred to by the authors as “tiredness”^3^). In this model, changes at a somatic level and/or within the hierarchical system’s circuitry in response to disease disrupts computational functions (which may involve various levels of the hierarchy) such that dyshomeostasis (error within the interoceptive model) is persistently experienced, engendering a perceived lack of control over bodily states which in turn fosters the conscious perception of fatigue. Importantly, in pathological fatigue, these disruptions affect the system in such a way that the detection of dyshomeostasis is not abated even through rest. Therefore, mastery over body states is not easily restored and the perception of fatigue endures. This is the fundamental difference between chronic, pathological experiences of fatigue and the acute feeling states incurred through physical activity. In the latter, prediction errors arising from metabolic responses to exercise can be effectively attenuated by action (i.e., rest), allowing the gradient of interoceptive “surprise” to turn negative and thus agency to be experienced through the restoration of homeostasis during a period of recovery.

However, Stephan and colleague’s description of acute symptoms of fatigue does not explore the dynamics of the inference-control loop during the homeostatic challenges imposed by prolonged physical exertion (Gabriel & Zierath, 2017; Jeukendrup et al., 2000; Joyner & Coyle, 2008). Thus, an explicit account of how the acute, transient symptoms of fatigue emerge in relation to protracted physical exertion remains absent. Here, we conform to the proposition forwarded by Stephan et al. (2016)—that fatigue arises from the metacognitive detection of persistent prediction error which reduces perceived capability of exerting effective control over the body (reduced self-efficacy)—and extend the metacognitive theory of dyshomeostasis to describe the changing metacognitive beliefs and inferential computations that may underlie the development of exertional fatigue.

## Transient Exertional Fatigue - A predictive processing model

According to Stephan et al. (2016), pathological fatigue may initially reflect an adaptive process, promoting rest and the conservation of energetic resources when effective actions to restore homeostasis are not perceived to be present. We extend this, contending that the experience of fatigue serves a similar function during acute physical exertion, in line with previous assertions (St Clair Gibson & Noakes, 2004). The selection of (goal-directed) actions may be devolved to an inferential issue, whereby actions and their predicted outcomes are chosen for the agent to ultimately frequent predictable states long-term(Friston, 2009; Friston et al., 2014). Importantly, this may involve predicting the consequences of actions across different temporal scales and contexts (Pezzulo et al., 2018). In some instances, the active experience of uncertain states may be tolerated if it helps guide future behaviour towards the minimization of prediction error (e.g., exploitation versus exploration; Schwartenbeck et al., 2013). However, for goal-directed actions to be maintained, the precision of the driving high-level beliefs must be high in order to dominate posterior beliefs, maintain attention and enable action (Pezzulo et al., 2015). However, all physical behaviour involves energetic expenditure, providing an ever-changing homeostatic context which must also be predicted within the transition towards the said outcome. The integration of salient, motivational information (e.g., bodily states) informs inferential processes that influence the selection of control policies in the pursuit of goal states (Pezzulo et al., 2018). Accordingly, for action to continue in any given form, interoceptive prediction error (and effects on control circuits) must be suppressed. However, the act of doing so, through attentional processes^4^ or lower-level perceptual updates, indicates that initial predictions did not provide a good fit to the sensory data.

**Fig. 2:**
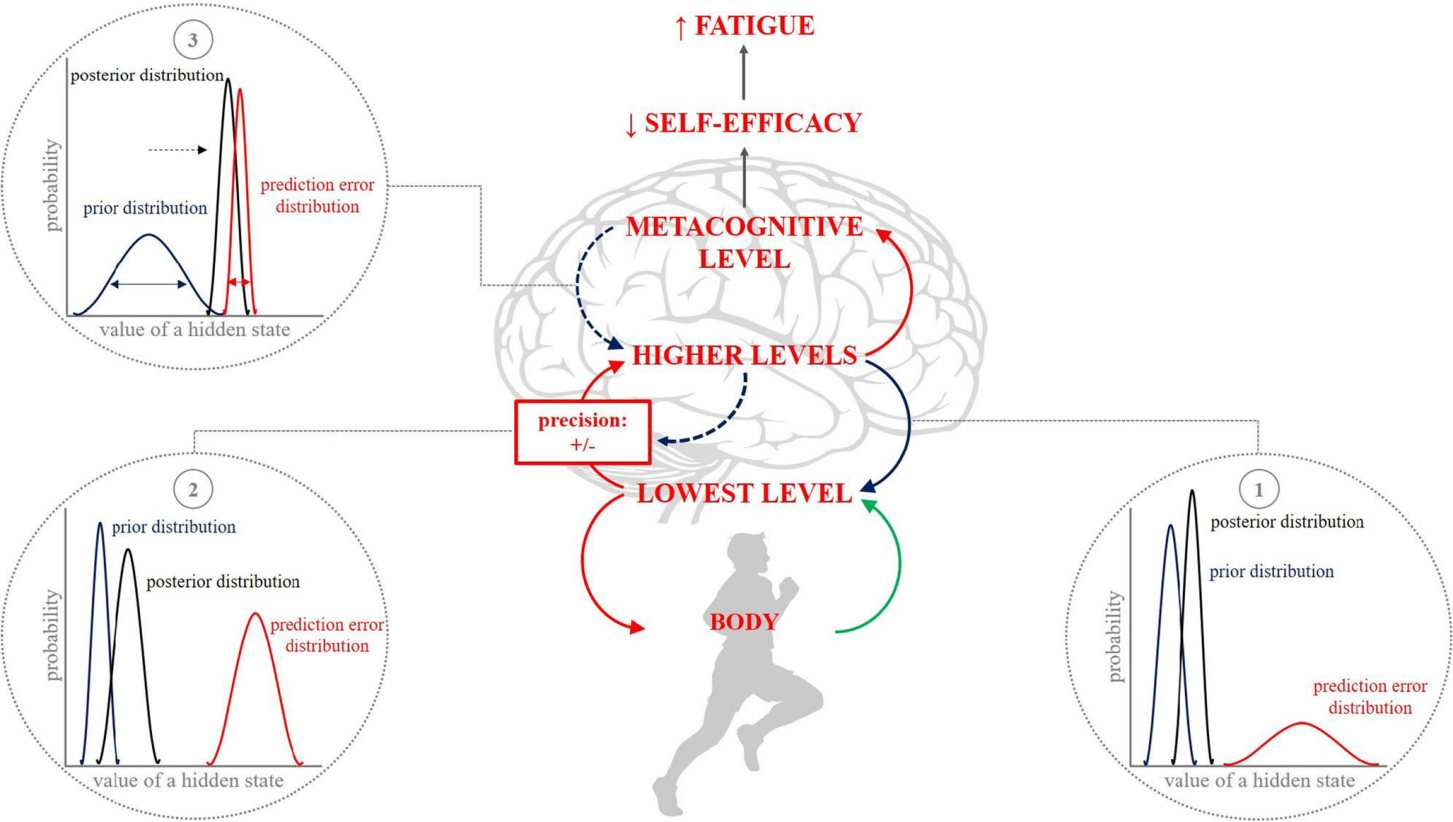
**Predictive processing framework underlying the emergence of exertional fatigue.** The engagement of protracted physical exertion requires internal models to accurately anticipate the sensory states that will be encountered to have the body reside within a (predictable) limited range of states that will sustain it biological integrity (*i.e.* maintain homeostasis). The subjective perception of fatigue may serve an adaptive function representing the ability of internal models to predict transition states during the pursuit of temporally distal goal states. **(1)** Under resting conditions or even low-intensity(physical) exertion, evidence of sensory states (green arrow) may be predictable (i.e., black posterior distribution dominated by blue prior beliefs). This may see the minimization of prediction error predominated by (autonomic) reflexes at the lowest level of the hierarchy. **(2)** However, as demands increase, and internal conditions becomes more unstable, physiological perturbations may be associated with greater prediction errors. Increasing strength of the prediction error (i.e., red distribution curve) may force error to ascend further up the levels of the hierarchy, necessitating deeper explanation, increasing its influence on posterior probabilities. This may generate attentional changes or perceptual updates across these lower levels. Yet importantly, as goal-directed action (i.e., physical exercise) is driven by higher-level beliefs, it may continue if the precision of these distal goal beliefs enables it to dominate prior updates and therefore contextualise the levels beneath (i.e., posterior distribution still dominated by prior beliefs). Estimation of the precision of beliefs is inferred in a separate stream (here simply represented by the grey circle). **(3)** Across time, the performance of the model’s overall prediction of the transition of states within goal pursuit is monitored by a metacognitive layer. Persistent detection of error within the hierarchy signals an inability to exert effective (allostatic) control of internal states during the pursuit of (longer term) goal states. This signals that the model may provide bad predictions about the present and, importantly, future condition(s) of the body. This perceived lack of control over bodily states undermines allostatic control self-efficacy, which is experienced as the subjective feeling of exertional fatigue. Computationally, the emergence of fatigue may be associated with declining precision estimates afforded to predictions driving goal-directed behaviour, signalling increasing uncertainty within the model and weakening the influence of priors on posterior beliefs (dashed blue line). The development of exertional fatigue is progressive, thus lower precision beliefs concerning goal-directed predictions result in greater prediction error throughout the levels of the hierarchy, which further undermine control capabilities during goal pursuit. Eventually, changing precision beliefs will see prediction error cause high-level, goal-directed beliefs to be updated (i.e., shift in posterior distribution towards prediction error) which may shift control priorities toward the resolution of more immediate prediction error. This may be achieved through action (i.e., rest). Over time, rest restores self-efficacy in ones' ability exert control over bodily states through the experience of agency (i.e., accurate predictions) in the restoration of homeostasis. Fatigue is therefore alleviated. However importantly, due to the significant challenge to model evidence encountered, restoration of perceived mastery of the body and homeostasis may be protracted. This is because precision estimates of predictions may be so low that prediction error is exacerbated during the recovery period. Therefore, the detection of accurate allostatic predictions may be strewn with prediction error which prolongs the subjective experience of fatigue. *Red arrows represent ascending prediction error, blue arrows represent descending predictions and green arrows represent ascending sensory evidence from the body. Dashed blue line represents effects on precision estimates*

The continued need to suppress prediction error in the pursuit of goals is the important extension of the proposal outlined by Stephan et al. (2016), forming the basis of metacognitive inference dynamics leading to the experience of fatigue emerging from acute (physical) exertion. We propose that the suppression of prediction error at all lower-﻿levels during the maintenance of goal-directed action driven by higher levels, is subject to the same metacognitive appraisal as described in the conceptualisation of pathological fatigue. Persistent experience of error within hierarchical models undermines (short-term) control mastery, reducing self-efficacy and is experienced phenomenologically as fatigue. Metacognition within predictive processing may be ascribed computationally to beliefs about the precision of priors (Friston et al., 2013). The metacognitive experience of fatigue may therefore be associated with an increasing uncertainty in expected sensory states, reflecting low precision beliefs cascading down from high to low levels of the hierarchy. Low precision at higher, temporally-distal, and more abstract levels of prediction may be unable to contextualise prediction error effectively. Eventually over time, this decline in precision beliefs may be such that there is a shift in the dominant control of behaviour towards lower levels, which attempt to reconcile more immediate differences between predictions and prediction errors (Pezzulo et al., 2015; Fig. 2).

To illustrate this hypothesis, we provide an example based on the experience of fatigue during sustained physical exercise. Once the decision to engage with exercise has been made, the brain must predict not only the proprioceptive consequences of action, interactions with the social and physical environment, but also the homeostatic consequences of the transition towards the intended goal state, in order to maintain biological integrity. This is obviously challenging, since protracted whole-body exercise incurs a vast array of ever-changing processes across a range of biological systems (Hawley et al., 2014); the strength of which may exceed the control capable by simple reflexes at the lowest level and therefore competes for attention and higher-level control (Fig. 2). To continue towards goals (*e.g.* persist with the current exercise task), precise, high-level beliefs must be maintained. Action (*i.e.* reducing intensity) offers a means to minimize interoceptive prediction errors during goal pursuit but in certain instances this may directly challenge the probability of the expected or intended goal state being reached.

Alternately, changes in attention and perceptual updates arising from prediction error minimization at lower levels may serve to enable goal-directed action to continue in a given form (e.g., speed). This may however, come at the expense of incurring further prediction error further on within a new inferential cycle. Regular and continued detection of error and the need for ensuing updates to maintain action increases evidence against initial model predictions and the capacity to effectively control the body’s states during exercise. As such, self-efficacy in ones' capacity to experience future goal states whilst controlling homeostatic integrity in this transition is undermined and experienced consciously as fatigue. Reduced confidence propagates, top-down, through the hierarchy, thus higher goal beliefs increasingly lose their ability to predict future outcomes and contextualise lower-level prediction errors. At this point, a shift to lower-level controllers may attempt to restore self-efficacy by minimizing the experience of prediction error on more immediate timescales (Pezzulo et al., 2015). That is, we are forced to slow down or stop altogether.

Minimization of interoceptive prediction errors may not be immediate (e.g., replenishment of resting muscle glycogen stores can take many hours following exercise; Casey et al., 1995), therefore proprioceptive predictions^5^ may potentially be used as proxies for predicting the states which will eventually see interoceptive prediction error minimized (Critchley & Garfinkel, 2017; Pezzulo et al., 2018). By slowing down or stopping all together, the minimization of proprioceptive (and prospective interoceptive) prediction errors signals conditions that will see the restoration of homeostasis, indicating a level of control over bodily states which serves to alleviate fatigue (Stephan et al., 2016). However, the restoration of control self-efficacy is not immediate. Indeed, allostatic predictions used to maintain homeostatic set-points likely function across different biological systems under their own individual time constants (Stephan et al., 2016), which may see the experience of dyshomeostasis prolonged during rest. In addition, incurred deficits in allostatic self-efficacy and the subsequent decline in the precision afforded to control predictions (the proposed hallmark of fatigue) may underscore the protracted experience of fatigue post-exertion even when homeostatic balance has been restored. That is, increased sensitivity to prediction error during recovery (i.e., rest) may be maintained due to the loss of precision afforded to control predictions. It is possible that full alleviation of the conscious experience of fatigue may require the detection of a similar series of events to that through which it developed (i.e., protracted experience of accurate predictions related to interoceptive states). If this is the case, the experience of fatigue may urge us to stop in order to restore confidence in allostatic control beliefs and rest may represent the most effective context in which this may occur.

## Neuroanatomy of Interoceptive Monitoring During Physical Exertion – Neurophysiological evidence

The neuroanatomical circuits supporting interoception and homeostasis have been extensively described (Craig, 2002, 2003; Critchley & Harrison, 2013; Damasio & Carvalho, 2013) and incorporated within models of interoceptive inference and allostatic control (Barrett & Simmons, 2015; Gu et al., 2013; Stephan et al., 2016). Stephan et al. (2016) propose an additional metacognitive layer within established interoceptive pathways which monitors interoceptive prediction performance and is responsible for the emergence of fatigue. Putative structures include visceromotor regions, such as the anterior insula cortex (AIC) and anterior cingulate cortex (ACC)(Craig, 2002)and/or portions of the anterior prefrontal cortex (aPFC)(Fleming & Dolan, 2012). In support of the former, the experience of fatigue has been shown to correlate with increased activity within visceromotor regions following an acute interoceptive (e.g., inflammation) challenge (Harrison et al., 2009, 2015). The dense connectivity of the AIC and ACC has seen them identified as part of the brain’s “rich-club,” enabling communication across diverse networks (e.g., default and salience) and facilitating the representation of the body and allostatic control as integral component of psychobehavioural functions (Barrett & Simmons, 2015; Kleckner et al., 2017).

Strikingly, studies of acute physical and cognitive exertion have identified a similar network involving the AIC, ACC, and portions of the dorsal lateral PFC (dlPFC), activated with demanding activity across both domains (see Müller & Apps, 2019 for summary). This network was implicated in the development of fatigue, assigned specifically with the monitoring of neural-responses within task-specific regions and the computation of the cost/benefits of continued action (Müller & Apps, 2019). Insula activity has also been shown to parallel increasing physiological demands in response to greater exercise intensities and durations (Williamson et al., 1999), with pronounced mid/AIC activation associated with the point of task failure (Hilty, Jäncke, et al., 2011a). Enhanced synchronisation of insula and primary motor cortex (M1) activity also has been observed toward the end of volitional exercise (Hilty, Langer, et al., 2011b), which may reflect the influence of the dynamically changing conditions of the body on selected control (action) policies. Indeed, one plausible hypothesis, similar to that proposed recently by McMorris et al. (2018), is that AIC is responsible for generating an awareness of the performance of interoceptive predictions and allostatic control during exercise, and therefore fatigue. The representation of allostatic control performance may be continuously influenced by inputs from the dlPFC, which may signal the level of uncertainty within (longer-term) predictions. The ACC is believed to play an important role in deciding between actions during physical exertion (Lutz, 2018) and thus may be involved in encoding the estimated precision of competing predictions and prediction errors based on inputs received regarding the performance of interoceptive predictions (see also Craig, 2009). Precision dynamics arbitrate between the dominant controllers within the hierarchy in pursuit of the suppression of (long-term) prediction error (Pezzulo et al., 2015), with the encoding of precision assigned to neuromodulatory systems (e.g., dopamine; Friston et al., 2012). It has been proposed that the development of exertional fatigue may coincide with changes in the firing of neuromodulatory neurons within the (lateral) PFC, modulating motivation and choice based on the integration of interoceptive and reward pathways (McMorris et al., 2018). The current proposal holds that changes in neuromodulatory control across various regions of the brain reflects the optimisation of precision across the hierarchy to minimize prediction error, and fatigue is associated with a progressive reduction in precision estimates of high-level predictions driving action.

## Testing the Model – Current empirical support and future hypotheses

The core proposition of the proposed model contends that detection of repeated prediction error within interoceptive circuits during acute bouts of physical exertion undermines control efficacy beliefs, reductions in which provide the foundation of the perception of fatigue. Several lines of evidence in the existing literature support these central assertions and provide the basis of future investigations.

Multiple studies have investigated the association between self-efficacy, emotion (Focht et al., 2007; Magnan et al., 2013; McAuley et al., 1999), and behavioural responses to acute physical exertion (Halper & Vancouver, 2016; Hutchinson et al., 2008; McAuley & Courneya, 1992; Weinberg et al., 1981). Perceived capacity to successfully engage in further physical exertion has been demonstrated to both increase (Blacklock et al., 2010; Katula et al., 1999) and decrease (Focht et al., 2007; Katula et al., 1999; Welch et al., 2010) in response to acute bouts of exercise. The direction of this effect appears to be associated with the intensity of the bout (Blacklock et al., 2010; Katula et al., 1999). Deterioration in performance self-efficacy has been demonstrated at intensities at which a change in the metabolic environment is evident (Welch et al., 2010) and importantly, this decrement has been associated with the phenomenological experience of fatigue (Focht et al., 2007). These findings provide support for the proposition that physiological perturbations evoked by demanding physical exertion impact perceived efficacy which may be the origin for the acute, transient experience of exertional fatigue. However, future research is required to empirically establish whether unstable metabolic responses during exercise lead directly to a deterioration in self-efficacy and to confirm the temporal association between changes in perceived efficacy and the phenomenological experience of fatigue during physical exertion. Weakening of self-efficacy is proposed to evolve from continued metacognitive detection of prediction error (Stephan et al., 2016). Presently, few studies have directly examined the effect of physical exertion on metacognitive function (Palmer et al., 2019), despite recognition that it is a process fundamental to self-regulation during prolonged physical exertion (Brick et al., 2015; Brick et al., 2020; see Brick et al., 2016). Future examination of the metacognitive basis of exertional fatigue under the present proposal could see the use of a combination of metacognitive assessments alongside the modelling of precision estimates from behavioural responses (Hezemans et al., 2020; Wolpe et al., 2014). Interestingly, using this behavioural modelling approach, apathy—a construct often correlated with fatigue—has recently been associated with a low precision-weighting of performance predictions (Hezemans et al., 2020).

It is unclear whether potential reductions in self-efficacy relate to confidence in one’s ability to control or accurately perceive viscerosensory information. Experimental manipulation of prediction error within interoceptive paradigms provides a suitable challenge. The exercise science literature is replete with experimental interventions designed to manipulate physical or perceived states during exercise, with a predominant focus on altering interoceptive-associated feedback (Amann et al., 2009; Castle et al., 2012; Hartman et al., 2019; Iodice et al., 2019). However, it is important to note for future investigations that under the current framework, perception is understood only when descending beliefs and ascending sensory evidence are considered in conjunction. To highlight this point, recent evidence demonstrated that perception of a weak, exteroceptive stimulus was influenced by its relative timing within the cardiac cycle with effects of presumed heartbeat-event predictions observed throughout the somatosensory cortex (Al et al., 2020). It is expected that interoceptive self-efficacy will be most impaired when the divergence between expectations and physiological states is at its greatest.

In healthy populations, sustained contractions are perceived as more effortful when performed in a perceived state of fatigue, even in condition absent of any functional changes to the active muscles (Greenhouse-Tucknott et al., 2020). Similarly, effort perception during motor tasks has been associated with trait fatigue in clinical populations, such as stroke survivors, suffering from chronic fatigue (De Doncker et al., 2020)^6^. This may be explained within the proposed model as follows: lower precision estimates in prior beliefs at higher levels emerging with (exertional) fatigue (reflecting increasing uncertainty in model predictions) cause greater prediction error at lower levels. Perception of effort is associated with movement or action costs, reflecting high gain, or insufficient attenuation, of action-induced prediction error (Kuppuswamy, 2017), or more formally described under information theoretical principles, as the information cost of updating prior beliefs (Zénon et al., 2019). Therefore, the decline in precision estimates associated with fatigue form greater prediction error that generates a heightened perception of effort. What is not currently clear is whether, in healthy populations, the magnitude of fatigue experienced by an individual is directly proportional to the perceived effort during action. Further examination of the effect of increasing severity of fatigue on the perception of effort and the regulation of motor performance are warranted.

It is recognised that assessment of an individual’s interoceptive ability can span various dimensions, including the accuracy of one’s interoceptive judgments, self-reported confidence in those judgements and also insight into those judgements, i.e., metacognitive evaluation of the correspondence between objective accuracy and confidence in the accuracy of those judgements (Garfinkel et al., 2015; Garfinkel & Critchley, 2013; see also Khalsa et al., 2018). Despite its clear relevance to exertional fatigue and performance fatigability (McMorris et al., 2018; Robertson & Marino, 2016), studies on the effect of individuals’ (conscious and nonconscious) awareness of physiological states on perception and performance during physical exertion are sparse, conflicting and presently limited to measures of interoceptive accuracy (da Silva Machado et al., 2019; Herbert et al., 2007; Köteles et al., 2020). How an individual’s insight into representations of interoceptive signals influences acute physical exertion and the development of fatigue is currently unknown. Although yet to be empirically tested, it is possible that an individual’s interoceptive insight may change the relationship between a perceived state of fatigue and other perceptions arising from prediction error, such as the perception of effort, during physical exertion. That is, those who are more aware of the accuracy of their interoceptive judgments may experience smaller, relative reductions in precision beliefs under conditions of fatigue, therefore attenuating the rise in prediction error and the perception of effort. In line with this hypothesis, interoceptive insight has been shown to reduce one’s susceptibility to an exteroceptive manipulation of self-location (Bekrater-Bodmann et al., 2020), suggesting that precise higher-order representations of the body are effective at limiting the influence of (exteroceptive) prediction error on belief updates. Evaluation of how conscious insight into interoceptive representations of bodily shapes this relationship may help further define how metacognitive processes influence subjective experiences aroused during acute fatiguing exertion.

The neuroanatomical circuits supporting the development of pathological fatigue based on metacognitive detection of persistent interoceptive prediction-error have been proposed (see Stephan et al., 2016; “Neuroanatomy of interoceptive monitoring during physical exertion – neurophysiological evidence ” section). Functional resting-state connectivity and dynamic causal modelling (DCM) provides a potential means of establishing interactions between the nodes within this metacognitive interoception-network in response to fatigue (Stephan et al., 2016). Similar methods have recently been used to quantify changes in effective connectivity with the development of fatigue during protracted cognitive tasks (Wylie et al., 2020) and have indicated abnormal inhibition between hemispheres as a factor in the development of persistent fatigue in people with stroke (Ondobaka et al., 2019). In the context of the present model, it may be expected that an increased connectivity between visceromotor and prefrontal regions of the putative interoceptive metacognition network and primary interoceptive cortex (posterior insula cortex) (Barrett & Simmons, 2015) occurs as participants monitor the presence of dyshomeostasis during physical exertion. However, whether changes in effective connectivity between key nodes is associated with the development of exertional fatigue has yet to be elucidated.

## Concluding Remarks

In this perspective article, we draw upon recent propositions for the computational basis of pathological fatigue and extend this framework to provide an account of the subjective experience of fatigue evoked through acute exertion. Fundamentally, whether developing in response to pathology or as an acute, transient state during physical exertion, the experience of fatigue is a product of inference associated with an increased uncertainty in one’s capacity to accurately predict and control sensory states. This framework is fundamentally entrenched within current conceptualisations of how the brain works. We hope that with this article we have highlighted and indeed emphasised the potential of predictive processing to provide a common framework for the study of fatigue across health and disease.

## Footnotes

1 As described by Kuppuswamy (2017), if fatigue is the product of inference it is unitary and thus the common separation of fatigue between physical and cognitive domains may be unnecessary. Accordingly, the same principles described here in the explanation of fatigue emerging through physical exertion may also account for fatigue symptoms incurred through prolonged, demanding cognitive activity, although the exact locus of the disturbance (i.e., the level within the hierarchical system) and the specific systems (e.g., interoception vs. control) monitored may vary between domains (Müller & Apps, 2019).

2 The probabilistic representation of the causes of sensory data before observing the data.

3 We do not subscribe to the adopted terminology used by Stephan et al. (2016) in the description of feelings associated with chronic (i.e., fatigue) and acute (i.e., tiredness) experiences. Feelings of “tiredness” may be particularly problematic in the description of fatigue, because it also is used to describe a subjective state of sleepiness (Pigeon et al., 2003; Shen et al., 2006). Thus, tiredness may not accurately depict the subjective experiences reported after exertion (Leproult et al., 1997; Matsumoto et al., 2002). In response to demanding physical exertion, fatigue is a fundamental subjective experience.

4 We acknowledge the current understanding of volitional control of endogenous attention within predictive coding frameworks is not entirely clear (Metzinger, 2017).

5 An extension of the metacognitive theory of dyshomeostasis (Manjaly et al., 2019) proposes that metacognitive monitoring may not be exclusive to interoceptive circuits and may involve the detection a chronic mismatch between prediction and actual sensory evidence across different systems (e.g., proprioception; exteroception). In addition, interdependency between systems (Pezzulo et al., 2018) may see the persistent detection of error and development of fatigue permeate across different systems.

6 The authors propose that the experience of a high perception of effort drives the (trait) perception of fatigue in stroke survivors, rather than (state) fatigue driving elevated perceptions of effort (De Doncker et al., 2020). The model presented here suggests that the relationship between fatigue and effort may be circular; error is the basis of the perception of effort, which if persistently detected generates a feeling of fatigue. Changing precision beliefs associated with fatigue gives rise to greater error, which in turn elevates the perception of effort.
